# Antifungal Potential of Host Defense Peptide Mimetics in a Mouse Model of Disseminated Candidiasis

**DOI:** 10.3390/jof4010030

**Published:** 2018-02-27

**Authors:** Mobaswar Hossain Chowdhury, Lisa Kathleen Ryan, Kartikeya Cherabuddi, Katie B. Freeman, Damian G. Weaver, Jeffry C. Pelletier, Richard W. Scott, Gill Diamond

**Affiliations:** 1 Department of Oral Biology, University of Florida College of Dentistry, 1600 SW Archer Road, Gainesville, FL 32610, USA; masoomchowdhury@gmail.com; 2 Division of Infectious Diseases and Global Medicine, Department of Medicine, University of Florida College of Medicine, Gainesville, FL 32610, USA; lisa.ryan@medicine.ufl.edu (L.K.R.), Kartikeya.Cherabuddi@medicine.ufl.edu (K.C.); 3 Fox Chase Chemical Diversity Center, Inc., Pennsylvania Biotechnology Center, Doylestown, PA 18902, USA; kfreeman@fc-cdci.com (K.B.F.), dweaver@fc-cdci.com (D.G.W.), jpelletier@fc-cdci.com (J.C.P.), rscott@fc-cdci.com (R.W.S.)

**Keywords:** defensin, membrane-activity, *Candida*, peptide mimetics

## Abstract

Invasive candidiasis caused by *Candida albicans* and non-*albicans* *Candida* (NAC) present a serious disease threat. Although the echinocandins are recommended as the first line of antifungal drug class, resistance to these agents is beginning to emerge, demonstrating the need for new antifungal agents. Host defense peptides (HDP) exhibit potent antifungal activity, but as drugs they are difficult to manufacture efficiently, and they are often inactivated by serum proteins. HDP mimetics are low molecular weight non-peptide compounds that can alleviate these problems and were shown to be membrane-active against *C. albicans* and NAC. Here, we expand upon our previous works to describe the in vitro and in vivo activity of 11 new HDP mimetics that are active against *C. albicans* and NAC that are both sensitive and resistant to standard antifungal drugs. These compounds exhibit minimum inhibitory/fungicidal concentration (MIC/MFC) in the µg/mL range in the presence of serum and are inhibited by divalent cations. Rapid propidium iodide influx into the yeast cells following in vitro exposure suggested that these HDP mimetics were also membrane active. The lead compounds were able to kill *C. albicans* in an invasive candidiasis CD-1 mouse model with some mimetic candidates decreasing kidney burden by 3–4 logs after 24 h in a dose-dependent manner. The data encouraged further development of this new anti-fungal drug class for invasive candidiasis.

## 1. Introduction

Invasive candidiasis, including candidemia, is a serious fungal infection caused by *Candida albicans* and non-*albicans Candida* (NAC) species that can lead to mortality rates as high as 70%, depending on the population sampled [1]. The most common form of invasive candidiasis is blood infection, or candidemia, leading to disseminated candidiasis that can result in the infection of various tissues as well, resulting in infection of bone and liver, endocarditis, meningitis, pulmonary and splenic abscesses and endophthalmitis [2].

Despite the emergence of new antifungal therapies, particularly the echinocandins, the overall incidence of invasive candidiasis remains high, especially in populations susceptible to opportunistic infections such as elderly or immunosuppressed patients. However, due to underdetection and the difficulty of diagnosing non-candidemia candidiasis, the exact rate of invasive candidiasis is difficult to ascertain, and the incidence of candidemia varies depending on when and where the information was collected and the incidence denominator used in each study or surveillance [3]. Treatment options are complicated by the emergence of resistance to fluconazole and to echinocandins, the difficulty in diagnosis of invasive candidiasis, and the shift in infection from *C. albicans* to NAC spp. which are frequently more resistant. Many of these NAC spp. are preferentially selected using prophylactic anti-fungal therapy with fluconazole in the intensive care unit and in immunocompromised patients [4]. Fluconazole resistance in *C. albicans* is uncommon in the clinical setting, but common in NAC spp. especially with *C. glabrata* [5]*.* Echinocandins cannot be used for infections of the eye or urinary tract. For those infections, patients intolerant of fluconazole are only left with usage of Amphotericin with significantly higher toxicity. In addition, the relatively recent emergence of the multi-drug resistant *C. auris* is a concern. Thus, there is a great need for the development of new anti-fungal agents [3] which have activity in *C. albicans*, fluconazole sensitive and resistant NAC isolates.

One potential avenue to address this need is the development of host defense peptides (HDP). HDPs have broad-spectrum anti-microbial and immunomodulatory properties and develop antimicrobial resistance infrequently [6]. Examples of HDPs include magainin, α- and β-defensins, and cathelicidins such as LL-37. Attempts to exploit these desirable properties of HDPs as drugs have been thwarted by the cost of producing these peptides and mostly by the susceptibility to protease digestion and serum-binding properties in vivo, greatly diminishing their activity [7]. To circumvent these problems, the small synthetic, nonpeptidic oligomers were designed to adopt the amphiphilic secondary structures of HDPs and to exhibit potent and selective antimicrobial activity [8,9].

We have previously described two new, potent anti-fungal HDP mimetics that are active in vitro against both *Candida albicans* and NAC, even in the presence of 50% human serum, and are active against both the planktonic form and biofilms [10,11,12]. These HDP mimetics were prime candidates for anti-fungal drug development, for *C. albicans* grown long-term at sub-minimum inhibitory concentration (MIC) did not develop resistance to these mimetics [11] and they appeared to not affect commensal oral bacteria or biofilms [10]. These drugs also are active in vivo, both in an invasive candidiasis model and in an oral candidiasis model [12,13]. 

**Figure 1:**
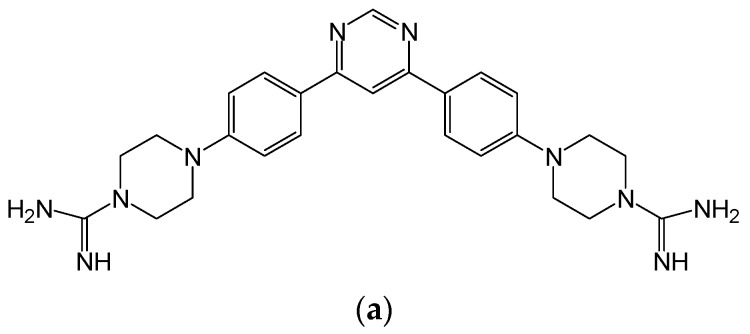

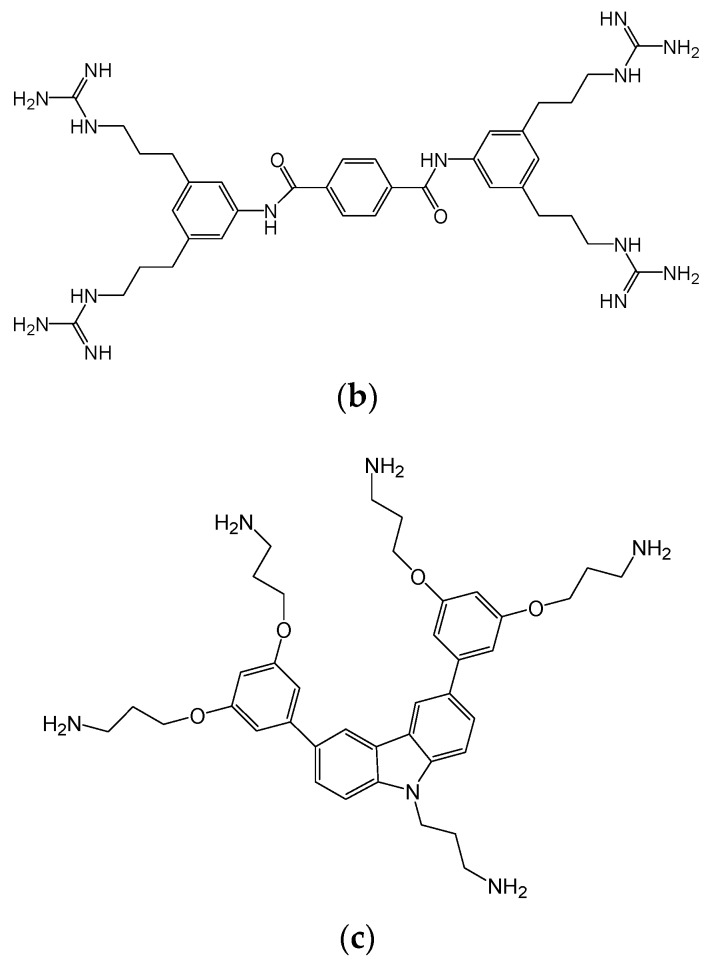
Representative compound structures. Structures of Compounds **1** (**a**), **2** (**b**) and **4** (**c**), corresponding to triacyl, bis-amide and tricyclic structures are shown.

In this study, we have described several new HDP mimetics with activity against *Candida* species. The compounds are analogs of Compounds **1**, **2** and **4** (see Figure 1 for structures) and have molecular weights ranging from 431 to 558 D. These are based on three different scaffolds, whose structure and activities have been previously described [14]. Several of these new HDP mimetics have potent anti-fungal activity against *Candida* both in vitro and in vivo.

## 2. Materials and Methods

### 2.1. Materials

Peptide mimetic compounds were received from Fox Chase Chemical Diversity Center, Pennsylvania Biotechnology Center, Doylestown, PA, USA. Compounds were dissolved in dimethyl sulfoxide (DMSO) (Sigma-Aldrich, St. Louis, MO, USA) at the stock concentration of 10 mg/mL and stored at −20 °C. Cyclophosphamide and fluconazole was purchased from Sigma-Aldrich, St. Louis, MO, USA. RPMI 1640 containing l-glutamine without bicarbonate, buffered to pH 7.0 with 0.165 morpholinepropanesulfonic acid (MOPS) buffer (Sigma-Aldrich, St. Louis, MO, USA).

#### 2.1.1. Yeast Strains

A standard invasive candidiasis strain of *C. albicans* SC5314 was used for peptide mimetic compounds screening. Non-*albicans Candida* species (NAC) *C. dubliniensis* (NCPF3949), *C. glabrata* (ATCC 90030), *C. krusei* (ATCC 6258), *C. parapsilosis* (ATCC 22019) and *C. tropicalis (*ATCC 750) (obtained from the laboratory of David Perlin, PHRI/Rutgers), were cultured on YPD (1% yeast extract, 2% peptone, 2% dextrose, pH 5.7) agar at 37°C and were used for all experimental studies. Fluconazole resistant clinical isolates of *C. glabrata* from five different patients and *C. tropicalis* from one patient were collected from the clinical microbiology laboratory, Shands Hospital, University of Florida, Gainesville, FL, USA.

#### 2.1.2. MIC and MFC Studies

*Candida albicans* SC5315, NAC species and fluconazole-resistant clinical isolates of invasive candidiasis strains of *C. glabrata* and *C. tropicalis* were used for minimum inhibitory concentration (MIC) and minimum fungicidal concentration (MFC) studies of lead compounds. MICs were determined using the Clinical and Laboratory Standards Institute (CLSI) standard broth microdilution method [12]. Suspensions of *C. albicans* were prepared by measuring optical density at 595 nm (OD_595_) of 1.0 in phosphate buffered saline (PBS). HDP mimetic compounds were diluted in 50 µL RPMI/MOPS cell culture medium in a 96-well plate (tissue culture treated; Costar, Corning Incorporated, Corning, NY, USA). To each well, a suspension (50 µL) of *Candida* was added, and then plate was incubated at 37 °C in a humidified chamber for a period of 24 h. To determine the MFC, a sample (100 µL) from the well defined as having the MIC and the wells with other three higher concentrations were plated onto YPD agar. *Candida* colonies were observed after 24 h. The MFC is defined as the lowest concentration of compound at which no colonies are observed. All MIC and MFC assays were performed in duplicate.

To assess the effect of a divalent cations on the electrostatic interaction between mimetics and cell membrane of yeast, we used calcium chloride at concentrations of 2, 5, 7.5 and 10 µM. The study was conducted using both RPMI/MOPS cell culture medium and Muller Hinton (MH) broth. At each concentration of this cation, the MIC of mimetics were determined by the method described above.

#### 2.1.3. Mammalian Cell Cytotoxicity Assay

Cytotoxicity (50% effective concentration (EC_50_)) was determined against mouse 3T3 fibroblasts (ATCC CRL-1658), OKF6/TERT-1 cells (oral keratinocytes) [12], and human transformed liver HepG2 cells (ATCC HB-8065), using an MTS viability assay (Promega, Fitchburg, WI, USA). Growth medium was replaced with medium without serum, and eight 2-fold dilutions of compound were added. Following incubation for 1 h at 37 °C, compounds were removed, and medium containing serum was returned. Viability was determined using an MTS viability assay (CellTiter 96 aqueous nonradioactive cell proliferation assay, Promega).

#### 2.1.4. Hemolysis Assay

Peptide mimetic compounds were tested in duplicate using a hemolysis assay previously published [15,16]. Briefly, two-fold serial dilutions starting at 2 mg/mL in 150 mM NaCl (saline), 20% DMA (Alfa Aesar, Ward Hill, MA, US), or 10% kleptose in saline. 3% saponin (Fluka) in saline was used as a positive control. Each dilution of each compound (25 µL) was plated in a 96-well round bottomed plate and 25 µL of whole human blood (BIORECLAMATION, Westbury, NY, USA) was added to each sample and mixed for 10 second. The blood was Type O, from 2 males and 2 females, collected with potassium EDTA to prevent clotting. Plates were incubated on a shaker for 30 min at 37 °C, then centrifuged at 600× *g* for 5 min. 5 µL of the supernatant was transferred to a 96-well flat-bottomed plate with 200 µL H_2_O in each well and mixed for 5 min. Absorbance was read at 405 nm (Spectramax 250, Molecular Devices, San Jose, CA, USA). Percent hemolysis was then calculated as: 

P = (OD_405_ (PMX compound) – OD_405_ (control))/(OD_405_ (Saponin) – OD_405_ (control))

Curves were fitted to report an HC_50_ (50% hemolysis) value for each compound:

P(C_p_) = 1/(1 + (K/C_p_)^n^)

#### 2.1.5. Yeast Membrane Permeability Assessment

*C. albicans* was suspended in RPMI/MOPS media to a total concentration of 2 × 10^6^ cfu/mL. One ml aliquots of the *C. albicans* suspension were treated with each mimetic compound at a concentration of 32 µg/mL, using 95% ethanol as positive control. Samples were incubated with the mimetics at 30 °C for 0, 5, 15 and 30 min. After treatment, samples were centrifuged at 5000 rpm for 5 min. Supernatants were removed and pellets were washed twice with 1 mL of PBS. Lastly, after the supernatant was removed, 50 µL of PI (0.5 µg/mL) was added and mixed via inversion for 15 min, and the pellet was resuspended in 500 µL of PBS. Samples were analyzed by flow cytometry (FACSCalibur, Becton Dickinson, Immunocytometry Systems, San Jose, CA, USA) to assess percent uptake of PI.

### 2.2. Efficacy of HDP Mimetics in vivo

#### 2.2.1. Mice

Female CD-1 mice aged from 4 to 6 weeks (weight, 20–22 g) were used in this study. Mice were purchased from Charles River, Wilmington, MA, USA. All mice were housed in a barrier facility in the Biomedical Science Building Animal Facility, University of Florida. Animals were fed a sterilized standard rodent diet and drinking sterilized water *ad libitum*. Mice were acclimatized to the animal house environment for 7 days prior to use in experiments.

#### 2.2.2. *Candida* Strains and Inoculum Preparation

*C. albicans* SC5314 were grown in YPD broth (Difco laboratories, Detroit, MI, USA) and stock cultures were stored at −80 °C. The frozen stocks were first grown on YPD plates and kept at 4 °C. Prior to each experiment, a stock inoculum suspension of *Candida* was prepared in YPD broth and incubated at 30 °C with shaking at 200 rpm for 12 h. The culture was washed twice with sterile 1X phosphate buffered saline (PBS), counted with a hemocytometer, and diluted with PBS to get the desired concentration of 3.5 × 10^5^ cfu/mL. 100 µL was injected intravenously (IV) into each mouse via the tail vein. The desired infection dose was confirmed by plating 10-fold dilutions of the inoculum on YPD plates, which were incubated at 37 °C for 24 h to yield viable counts (cfu) of yeast colonies [12].

#### 2.2.3. Drug Preparation

For the animal studies, mimetics were dissolved in 20% kleptose at a 2 mg/mL concentration and sonicated for 10 min. Cyclophosphamide (150 mg/kg in 10 mL/kg) was dissolved in sterile PBS. Fluconazole (5 mg/mL) was dissolved in 10% DMSO in sterile PBS.

#### 2.2.4. Determination of the Maximum Tolerated Dose (MTD)

Female CD-1 mice (Charles River Laboratories), 8–10 weeks old, >20 g were used to determine the MTD. Mice were housed individually in stainless steel, wire-bottom cages to minimize access to urine and feces and fed Harlan Teklad Certified Global Diet^®^ with tap water *ad libitum*. Mice were acclimated to the study room at least 3 days prior to dosing. Mice (3 per group) were dosed with a subcutaneous (SC) bolus injection beginning with 10 mg/kg of the test compound at a concentration of 2 mg/mL. Doses were lowered two-fold for subsequent groups to find the MTD. The dose volume was 5 mg/kg, calculated using the weight of the animal on the day of injection. There were 3 dose events, with 2 days between each event. Mice were observed for changes in appearance and behavior at 2 and 24 h following each injection and mice showing pronounced immediate effects were removed from the study. Body weights were obtained daily until the day of euthanization with a CO_2_ overdose. Any mortality during the study was also recorded. MTD data were obtained by Ricerca Biosciences, LLC, Concord, OH, USA. MTD of each HDP mimetic in CD-1 mice was determined and our studies utilized doses beginning with 0.5X MTD.

#### 2.2.5. Immunosuppressive Mouse Model of Invasive Candidiasis and Comparison of HDP Mimetic Efficacy at 50% MTD

Male CD-1 mice, 6–8 weeks old (20–22 g), were made neutropenic with two intraperitoneal (IP) injections of 150 mg/kg in 10 mL/kg cyclophosphamide four days and one day before inoculation [17]. Each animal was then inoculated by injecting 0.1 mL of 3.5 × 10^5^ cfu/mL *C. albicans* in the tail vein. Animals were divided into different groups (*n* = 5 per group). 2 h after *C. albicans* inoculation, mimetic compounds were injected subcutaneously (SC) at 0.5X of the MTD (determined by Charles River Laboratories as described). Fluconazole (20 mg/kg) was administered to another group by oral gavage at 2 h post inoculation, as a positive control for the experiments, to confirm the ability to quantify an effect on kidney burden. The animals were humanely killed by CO_2_ asphyxiation and both kidneys were collected from five mice of infected control group 2 h after infection and from the remaining control and drug-treated mice in the study at 24 h after infection. Two kidneys were weighed and combined in a sterile homogenizer tube/mouse. Ten mL sterile PBS was added to each tube and the contents uniformly homogenized with a tissue homogenizer (Ultra-TURRAX Tube Drive, IKA Works, Inc., Wilmington, NC, USA). Serial dilutions of the tissue homogenates were conducted, 0.1 mL aliquots were spread on YPD agar plates, and the plates incubated at 35 °C overnight. cfu/g kidneys were determined from colony counts.

#### 2.2.6. Single Injection Dose Response Study of HDP Mimetics

Various doses of each compound were administered as a single SC injection of compound at 2 h after infection and cfu/g kidney were determined at 24 h post infection. The cyclophosphamide induced CD-1 neutropenic mouse was used as described above. Mice were intravenously inoculated with 3.5 × 10^5^ cfu/mL *C. albicans* via the lateral tail vein. After 2 h of infection, a single SC injection of various doses (40, 20 and 10 mg/kg, body weight) of Compound **6** were given to mice (*n* = 5 per group) at 2 h post infection. A fluconazole treated group was also tested for comparison. At 24 h post infection, all animals were sacrificed and kidneys were harvested, and cfu/g kidney was determined.

#### 2.2.7. Comparison of a Single Dose with a Split Dose of HDP Mimetic

Groups of 5 mice were given 0.5 MTD of Compound **6**, either as a single bolus SC dose, or twice half of the 0.5 MTD dose 2 or 4 h apart. The compound was initially given 2 h following infection and kidney cfu was assessed 24 h following infection. Infection was given as above, with 3.4 × 10^4^ cfu/mouse injected IV into the dorsal tail vein.

### 2.3. Statistical Analysis

All graphic data are expressed as mean ± SD and were statistically analyzed by analysis of variance (ANOVA) using computer Prism software (Prism 5; GraphPad Software, Inc., San Diego, CA, USA). Burden difference between testing and control groups was assessed by post hoc analyses, using Dunnett’s multiple comparison test. *p* < 0.05 was considered statistically significant. The EC_50_ was calculated using GraphPad Prism software (nonlinear fit).

### 2.4. Ethical Statement

All animals were maintained in accordance with American Association for Accreditation of Laboratory Care criteria. The animal experiments were designed and conducted upon approval of the Institutional Animal Care and Use Committees (IACUC) (protocol number 201408371, 29 May 2014), University of Florida, USA and strictly in accordance with the guidelines of IACUC.

## 3. Results

### 3.1. In Vitro Efficacy of HDP Mimetics against Candida Species

To screen for potentially effective compounds, MIC/MFC assays were performed, with and without 50% human serum. Table 1 compares the in vitro activity of HDP mimetics regarding MIC, with and without 50% human serum, and MFC (without human serum) against *Candida albicans*, strain SC5314. Most compounds were also tested for activity against five non-*albicans* species (NAC): *C. tropicalis, C. parapsilosis, C. dublinensis, C. glabrata, and C. krusei.* All compounds showed broad activity against all the *Candida* species, with the MIC of Compounds **3**, **5**, **7** and **6** rising from 2 or 4 to 16 µg/mL with the addition of 50% human serum to the assay with *C. albicans*. We then tested Compound **6** against fluconazole-resistant NAC derived from clinical isolates from different patients with invasive candidiasis. In five patient isolates of fluconazole-resistant *C. glabrata*, the MIC was >64 µg/mL. However, with one isolate of fluconazole-resistant *C. tropicalis*, Compound **6** had an MIC of 4 µg/mL and an MFC of 32 µg/mL (Table 2).

Table 3 shows in vitro effects on mammalian cells, assaying for cytotoxicity and hemolysis. These parameters were variable among the HDP mimetics, but the cytotoxicity and hemolytic capacity was low. Compound **6** was chosen for further testing due to its lack of cytotoxicity and hemolysis (Table 3). The cytotoxicity EC_50_ on both 3T3 and HepG2 cells was >1398.6 µM and for hemolysis, the EC_50_ was 1331 µM. Furthermore, Compound **6** had a high maximum tolerated dose (MTD) of ≥40 mg/kg. Table 4 shows all MTD in CD-1 female mice for all compounds. The best tolerated compounds were Compounds **6** and **8**. However, Compound **8** had an EC_50_ of 390 µM for 3T3 cells and 249 µM for HepG2 cells, which was much lower than Compound **6**.

### 3.2. Membrane Effects of HDP Mimetics on Candida albicans

**Figure 2:**
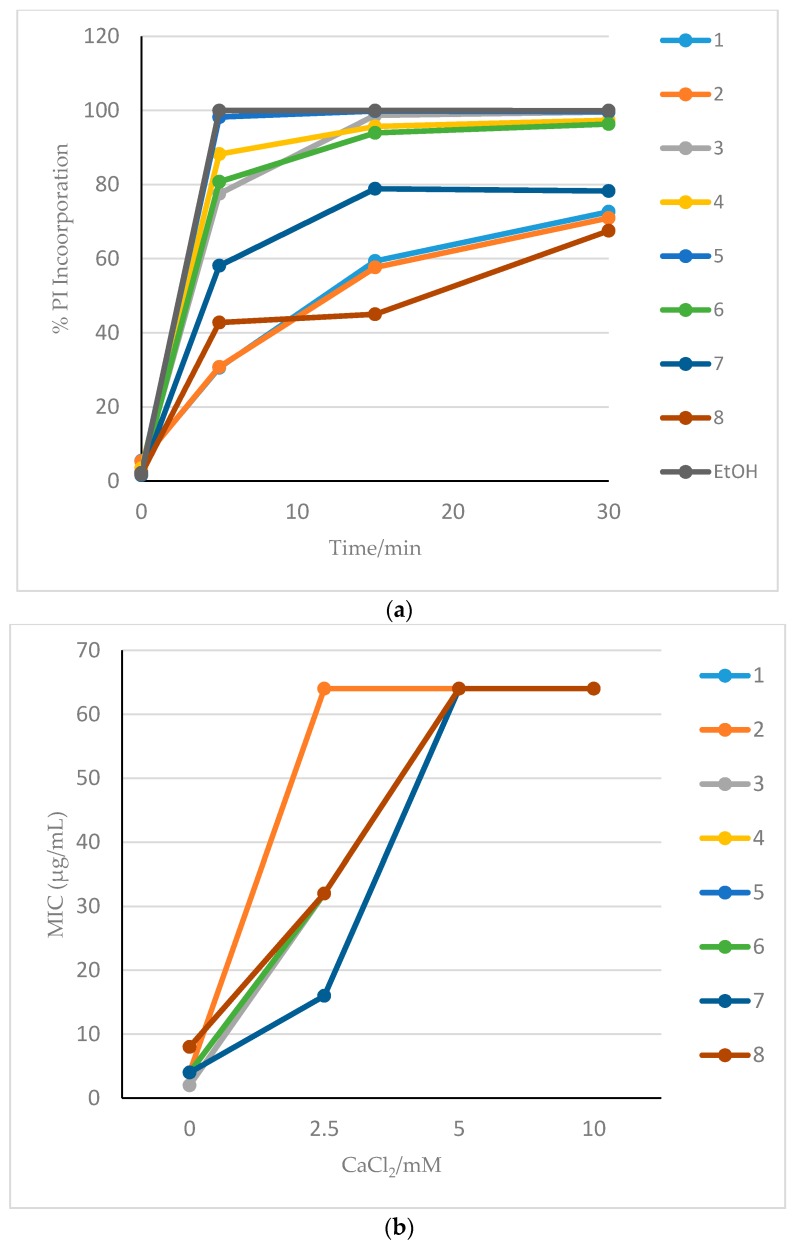
Membrane activity of potent HDP mimetics. (**a**) Propidium iodide (PI) incorporation in *Candida albicans* exposed to HDP mimetics. PI incorporation after 0–30 min treatments with 32 µg/mL of each HDP mimetic. 95% ethanol was used as a positive control, in which 100% PI entered the cell within 7 min. No PI was taken up in untreated yeast cells. (**b**) Calcium chloride inhibition of MIC of potent HDP mimetics. MIC assays were carried out in the presence of increasing concentrations of CaCl_2_.

We previously demonstrated that Compound **4** acts on *Candida albicans* by leading to rapid pore formation [13]. To determine whether these other compounds exhibit a similar mechanism, we quantified the propidium iodide (PI) incorporation after drug treatment. When 32 µg/mL PI was added to cells following the addition of each HDP mimetic to *C. albicans*, influx of PI occurred within 5 min and maximum incorporation of PI with all compounds occurred by 30 min, although each reached the maximum at different rates (Figure 2a). Treatment of *Candida* with Compounds **1**, **2**, **7** and **8** resulted in reaching maximum incorporation of PI in 30 min (the longest), while the most rapid membrane activity was observed with Compounds **3**, **4**, **5** and **6**.

A second aspect of the antifungal mechanism of Compound **4** is the initial binding to of the cationic compound with the anionic membrane. To determine whether the compounds assayed here exhibit a similar electrostatic interaction, we inhibited the activity with CaCl_2_ as a divalent cation. Figure 2b shows the effect of adding CaCl_2_ on the activity of eight HDP mimetics in the MIC assay using *C. albicans*. The divalent cation was able to inhibit the activity of all eight compounds on *C. albicans* at a CaCl_2_ concentration of 5.0 mM, raising the MIC to over 60 µg/mL. Adding CaCl_2_ to Compounds **2** and **3**, raised the MIC to >60 µg/mL at the lower concentration of 2.5 mM CaCl_2_.

### 3.3. Efficacy of HDP Mimetics in a Mouse Model of Invasive Candidiasis

**Figure 3:**
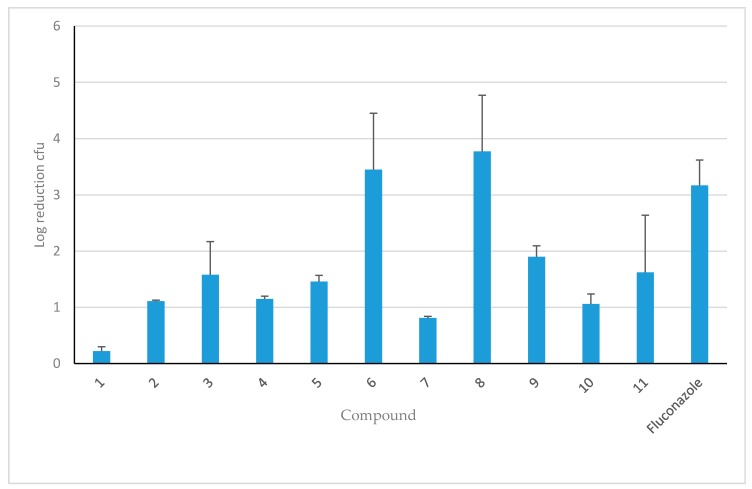
Comparison of HDP mimetic efficacy at 50% MTD. Mean log_10_ reduction of colony forming units (cfu) of *Candida albicans* following SC treatment with different HDP mimetics at 50% of the maximum tolerated dose (MTD) 2 h following IV infection with 3.5 × 10^4^ cfu/mouse for 24 h. For comparison, female Swiss CD.1 mice were gavaged 2 h after infection with 20 mg/kg fluconazole for 24 h, which caused a 3-log reduction in cfu. Groups were compared to a control group dosed with kleptose and infected with 3 × 10^4^ cfu *C. albicans* per mouse.

In an immunocompromised female CD-1 mouse model of candidiasis, with 3.4 × 10^4^ cfu/mouse injected IV, all HDP mimetics were compared by calculation of log_10_ reduction in kidney cfu 24 h after dosing SC with half of the MTD (Figure 3). Reduction in cfu were determined by calculating the log_10_ reduction in cfu for each treated mouse from the average of the 24 h untreated mice (*n* = 5). Data are shown in Figure 3 as the average of log_10_ reduction in cfu for each group (*n* = 5) ± SEM. The data for the fluconazole treatment (via gavage) were an average of the log_10_ reduction in cfu of four independent experiments and there was a 3-log reduction of *C. albicans* cfu in both kidneys using this standard treatment. Two HDP mimetics, Compounds **6** and **8**, appeared to have the greatest efficacy when mice were dosed with 40 mg/kg (assuming the MTD of >40 mg/kg is at least double, so 0.5X MTD is 40 mg/kg), giving a 3-log reduction in kidney burden (cfu/g). Other HDP mimetics reduced the kidney burden by two logs: Compounds **3**, **9** and **11**, each given SC at 0.5X MTD.

**Figure 4:**
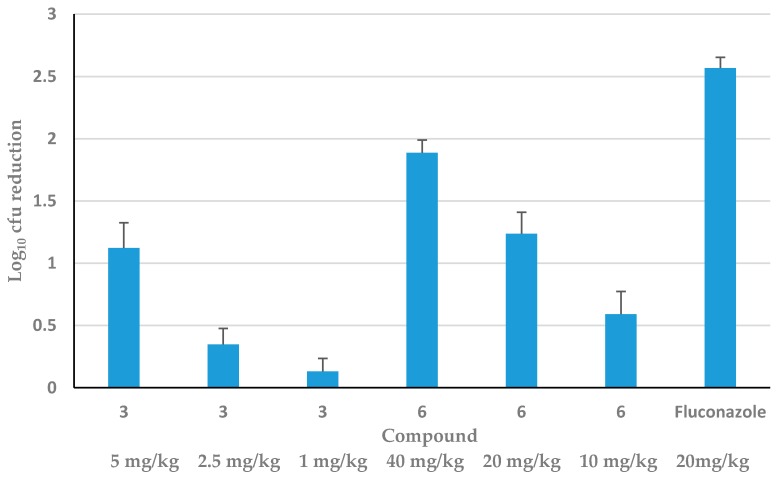
Dose-response of Compounds **3** and **6** in vivo. Female CD.1 mice (*n* = 5 per group) were infected IV with 3.5 × 10^4^ cfu/mouse before being treated 2 h later with a single SC injection of increasing concentrations of Compound **3** or **6** for 24 h. As a positive control, female Swiss CD.1 mice were also gavaged at 2 h with 20 mg/kg fluconazole for 24 h. Data are presented as log reduction in kidney burden (±SEM) from the mean cfu of untreated mice (*n* = 5). The dose-dependent reduction in kidney burden was significant (*p* < 0.05).

Considering toxicity and efficacy, two promising HDP mimetics were tested in vivo for dose-response. Figure 4 shows both the kidney burden (2 kidneys, cfu/g kidney) and the log reduction of *C. albicans* using three doses of Compounds **3** and **6**, each showing a dose-response. Compound **6** reduced cfu by two logs at the highest dose of 40 mg/kg to 0.5 log at the lowest dose of 10 mg/kg. Compound **3** was not as effective; it only reduced cfu by one log at the highest dose of 5 mg/kg (0.5X MTD) and 0.5 log at the next dose of 2.5 mg/kg, whereas the lowest dose of 1 mg/kg of **3** had almost no effect.

**Figure 5:**
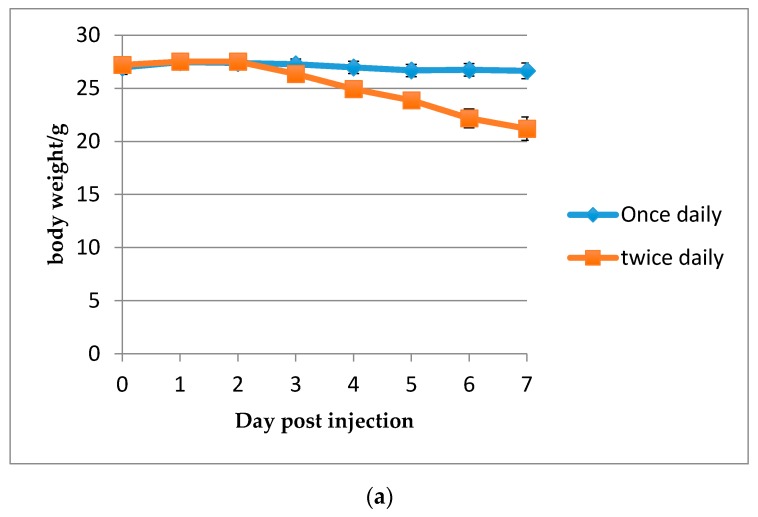

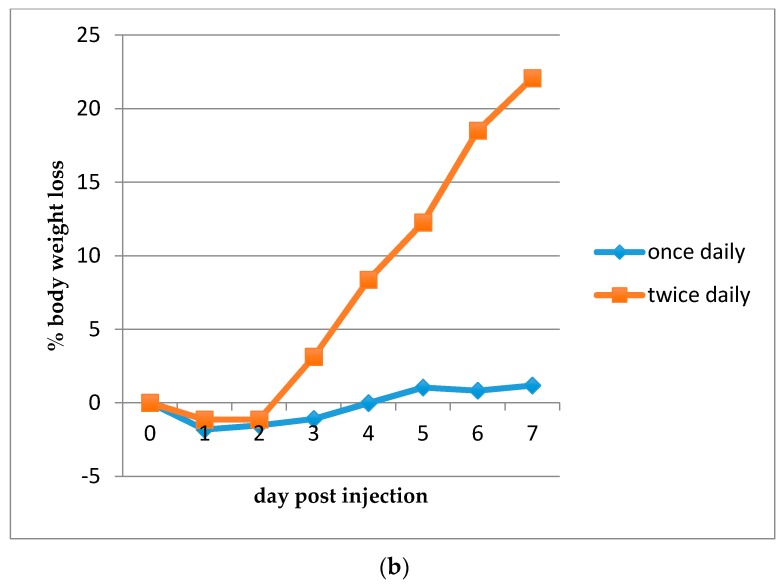
Effect of 20 mg/kg Compound **6** on body weight using two dosing regimens. (**a**) Body weight loss in grams following dosing once or twice, 6 h apart, for four consecutive days. (**b**) Percent body weight loss following dosing once or twice, 6 h apart, for four consecutive days. Two groups of 8-week old Female Swiss CD.1 mice were injected SC with 20 mg/kg of Compound **6** (*n* = 8). Mice were weighed daily for 7 days. Each point indicates mean ± SEM.

**Figure 6:**
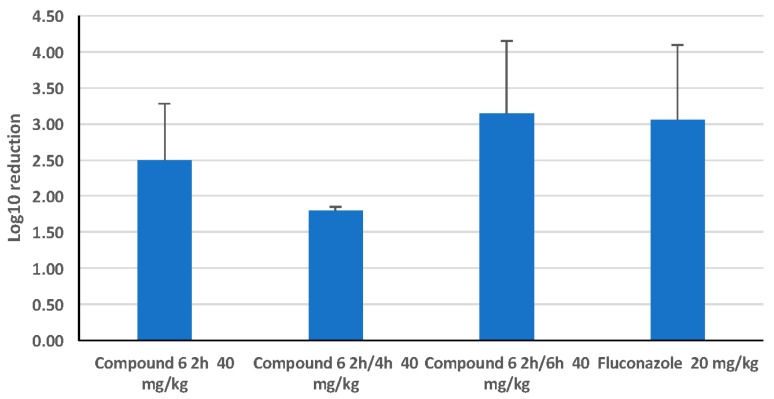
24-h treatment of *C. albicans* disseminated candidiasis with Compound **6** in single and split doses compared with fluconazole. Mean log_10_ reduction of colony forming units (cfu) of *Candida albicans* following SC treatment with Compound **6** at 50% of the maximum tolerated dose (MTD), 40 mg/kg, 2 h following IV infection with 3.5 × 10^4^ cfu/mouse for 24 h. Split doses of 20 mg/kg were given twice at 2 and 4 h and 2 and 6 h after infection. Groups were compared to a control group dosed with kleptose and infected with 3 × 10^4^ cfu *C. albicans* per mouse. For comparison, female Swiss CD.1 mice were gavaged 2 h after infection with 20 mg/kg fluconazole for 24 h.

Toxicity may be a concern with these compounds, so body weight was assessed following SC injection of Compound **6** without *Candida* (Figure 5). Body weight loss only occurred when injections of 20 mg/kg of Compound **6** were given twice per day. When split doses of 40 mg/kg of Compound **6** (20 mg/kg each) were given in the invasive candidiasis mouse model (Figure 6), reduction of kidney burden (cfu) remained consistent, with one single dose giving a log_10_ reduction of 2.5 and split doses giving statistically similar reductions in kidney burden.

## 4. Discussion

We have previously demonstrated that other HDP mimetics can kill *C. albicans* in the oral cavity and in a candidemia model of infection via a membrane-active mechanism [12,13]. Here, we have compared the activity and toxicity of these new HDP mimetic compounds (Table 1) both in vitro against in *C. albicans* and NAC (resistant and fluconazole-sensitive), with and without 50% human serum, and in vivo in CD-1 female mice systemically infected with *C. albicans* under two dosing regimens. Based on our results, HDP mimetics continue to be promising anti-fungal drug candidates both in vitro and in vivo. The compounds tested here appeared to have similar in vitro activity and minimal toxicity compared with the compounds described in our prior studies. We previously described the killing mechanism for Compound **4** as well as its in vitro and in vivo activity [13] and included this compound here for comparison. Although it is in a different class of anti-fungal agents, fluconazole (a triazole) was also included for comparison due to its ubiquitous usage in treatment of candidiasis. The MFC and MIC of these new HDP mimetics against *C. albicans* was comparable to both fluconazole and Compound **4**.

NAC spp. are becoming increasingly important isolates in invasive candidiasis [3]. Currently, in the USA, the proportion of *C. albicans* has dropped significantly to less than 50% of *Candida* infections, and *C. glabrata* accounts for more than a third of all candidemia isolates [3]. In Africa and Brazil, *C. parapsilosis* is almost as predominant as *C. albicans*, while in India and Pakistan, *C. tropicalis* is the predominant species [3]. Therefore, it was important to assess activity against NAC, especially *C. glabrata*. All HDP mimetic compounds exhibited low MICs for not only *C. albicans* but also *C. tropicalis, C. parapsilosis, C. dublinensis, C. glabrata* and *C. krusei*. Compound **6** exhibited activity (MIC) against a fluconazole-resistant isolate of *C. tropicalis* comparable to the MIC against fluconazole-sensitive *C. albicans* strain SC5314. Surprisingly, MICs against the other resistant clinical isolates exceeded 64 µg/mL, which demonstrated a reduced activity against fluconazole-resistant clinical isolates of *C. glabrata*. However, HDP mimetics that we have tested previously demonstrated that these compounds can effectively kill enchinocandin- and azole-resistant *C. albicans* and azole-resistant *C. krusei* in vitro, with an MIC of 0.5 and 1.0 µg/mL, and an MFC of 1.0 and 2.0 µg/mL, respectively [10]. This observation suggests that more in-depth structure-activity studies could reveal mechanisms of *Candida* resistance to these HDP mimetics, which would drive synthesis of more effective HDP mimetics against these organisms.

When *C. albicans* was tested, the presence of 50% serum did not greatly inhibit the MIC of any of the compounds, although there was slight variation in the MIC. Divalent cations were able to inhibit HDP mimetic activity, suggesting that the initial interaction of the cationic compounds is an electrostatic interaction with the anionic fungal membrane. Propidium iodide rapidly entered the *C. albicans* cells following addition of these drugs, suggesting the rapid creation of pores in the membrane after binding. The results were very similar between each HDP mimetic, although the mimetics had different rates of influx, suggesting that the compounds were membrane active and killed *Candida* in a similar manner to Compound **4**, which we reported in detail in a prior study [13]. Together, these data show that these new HDP mimetics are fungicidal based on membrane destruction and perforation of the cell membrane and that human serum does not inhibit this killing, so these HDP mimetics are suitable for use in vivo.

Toxicity is a concern among the anti-fungal drugs currently in use. The strategy of therapy against candidemia is to hit hard (use a high tolerable concentration) and hit quickly (kill organisms in 24 h), making toxicity a risk [1]. The least toxic are the echinocandins, which are now the recommended first-line empirical treatment of invasive candidiasis [18]. Resistance to the echinocandins is rare, but it does occur. For example, the resistance of *C. glabrata* and *C. auris* to echinocandins in the USA ranges between 0–4% and 7%, respectively [1,3]. Amphotericin B, which was prescribed often in the past against systemic infections, is now often utilized when resistance to azole drugs or echinocandins emerges or is observed. However, amphotericin B is at the high end of the spectrum of toxicity of anti-fungal drugs, so it is reserved for such extenuating circumstances, such as intolerance or limited availability of or resistance to other antifungals [18].

The HDP mimetics may be able to fill the need for an anti-fungal drug that has little toxicity and can be used as an alternative for fluconazole when it is not tolerated, especially when echinocandins cannot be used for invasive candidiasis affecting the eye or urinary tract. In our experiments, very low cytotoxicity and hematoxicity was observed. The least toxic compound both in vitro (considering hemolysis and cytotoxicity in two cell lines, 3T3 and HepG2) and in vivo (considering MTD) was Compound **6**. With this compound, body weight loss was not observed in our invasive candidiasis mouse model during a single subcutaneous injection. When Compound **6** was injected twice in a day at 20 mg/kg, however, body weight loss occurred without infection with *Candida*. In addition, no advantage in *Candida* killing was observed with twice a day versus once daily dosing. Further pharmacokinetic dosing studies are necessary to determine the dosing schedule and best route of administration of these HDP mimetics to get the most effective treatment.

The in vivo efficacy of the HDP mimetics varied from as little as a quarter of a log reduction (insignificant) to as much as a four-log reduction in *C. albicans* cfu at 0.5 MTD. Two representative HDP mimetics, Compounds **3** and **6**, demonstrated killing in a dose-dependent manner. However, Compound **3** had a lower MTD and ED_50_ in HepG2 and 3T3 cellular cytotoxicity than Compound **6**, making it less desirable as a drug candidate to further develop, even though the MIC for *C. albicans* and NAC was in a favorably low range. Furthermore, at 0.5 MTD given 2 h after infection, Compound **6** caused a four-log decrease in *C. albicans* cfu, comparable to the 3-log decrease seen with 20 mg/kg fluconazole gavage 2 h after infection, showing promising effects for further development. Also, the cytotoxicity of **3** was higher, even though the MTD was comparable to Compound **6**, and the MIC was the highest in the presence of 50% human serum. This shows that these HDP mimetics can be manipulated to give different characteristics for therapy. Thus, Compound **6** could serve as a reasonable alternative to fluconazole.

The immunosuppressed CD-1 model of invasive candidiasis we used here is adequate for testing a small number of compounds for their relative in vivo activity. However, newer in vivo imaging techniques [19], which allow for non-invasive quantification of infection and drug efficacy will allow the larger-scale testing of mimetics. We are currently testing these methods (Ryan et al., manuscript in preparation).

In conclusion, the HDP mimetics, even though they are not peptides, have many of the characteristics of the HDPs upon which they were designed: Broad-spectrum activity, low risk of developing resistance, membrane activity, and rapid fungicidal activity. Further development will be necessary to ensure that this new class of anti-fungal peptides will be utilized clinically.

## Figures and Tables

**Table 1:** MIC study of eleven HDP mimetics.

Compound	1	2	3	4	5	6	7	8	9	10	11	Fluconazole	Caspofungin
Series	Tri-aryl	Bis-amide	Tri-cyclic	Tri-cyclic	Bis-amide	Bis-amide	Bi-aryl	Bis-amide	Bis-amide	Bi-aryl	Bi-aryl		
Activity against *C. albicans* (μg/mL)													
MIC	4	4	2	4	4	4	4	8	>8	0.3	8	0.25	0.25
MFC	8	16	8	8	8	8	8	16	>8	0.78	ND		0.5
MIC+50% Human serum	4	4	16	4	16	16	16	32	>8	ND	>8		
NAC species MIC (μg/mL)													
*C. tropicalis*	4–8	8	4	2–4	2	4	8	4	2	ND	1		
*C. parapsilosis*	4–8	1	1	2	1	2	2	4	0.5	ND	0.25		
*C. dublinensis*	8	32	16	4	2	2	4	4	ND	ND	ND		
*C. glabrata*	4	8	2	2	4	4	4	8	>8	ND	1		
*C. krusei*	32	8	0.50	16	2	2	4	4	1	ND	4		

ND, not determined.

**Table 2:** MIC/MFC of Compound **6** against fluconazole- resistant NAC clinical isolates compared with non-resistant *Candida albicans*.

Fluconazole-resistant NAC Derived from Clinical Isolates from Different Patients: TG-*Candida glabrata*, CT-*Candida tropicalis*	MIC (μg/mL)	MFC(μg/mL)
TG-1	>64	>64
TG-3	>64	>64
TG-4	>64	>64
TG-5	>64	>64
TG-6	>64	>64
CT-2	8	32
*Candida albicans* SC5314	4	8

**Table 3:** Cytotoxicity and hemolytic activity of HDP mimetics.

Compounds	Cytotoxicity 3T3 EC_50_ (µM)	Cytotoxicity HepG2 EC_50_ (µM)	Hemolysis EC_50_ (µM)
**1**	311	ND	>1792
**2**	358	ND	>1164
**3**	478	137	396.2
**4**	436	ND	>1170
**5**	891	315.1	1453.5
**6**	>1398.6	>1398.6	1331
**7**	>788.6	>788.6	>1577.3
**8**	390	249	>1272
**9**	725	1456	1173
**10**	>1445	>1445	>1445
**11**	>1518	>1518	>1517

**Table 4:** Maximum tolerated dose (MTD) of HDP mimetics in female Swiss CD.1 mice.

Compounds	1	2	3	4	5	6	7	8	9	10	11
MTD (mg/kg free base)	10	10	10	20	5	≥40	20	≥40	≥40	10	10

## References

[1] Bassetti M., Righi E., Montravers P., Cornely O.A. (2018). What has changed in the treatment of invasive candidiasis? A look at the past 10 years and ahead. J. Antimicrob. Chemother..

[2] Kullberg B.J., Arendrup M.C. (2015). Invasive Candidiasis. N. Engl. J. Med..

[3] Lamoth F., Lockhart S.R., Berkow E.L., Calandra T. (2018). Changes in the epidemiological landscape of invasive candidiasis. J. Antimicrob. Chemother..

[4] Pea F., Lewis R.E. (2018). Overview of antifungal dosing in invasive candidiasis. J. Antimicrob. Chemother..

[5] Whaley S.G., Berkow E.L., Rybak J.M., Nishimoto A.T., Barker K.S., Rogers P.D. (2016). Azole Antifungal Resistance in *Candida albicans* and Emerging Non-*albicans Candida* Species. Front. Microbiol..

[6] Diamond G., Beckloff N., Weinberg A., Kisich K.O. (2009). The roles of antimicrobial peptides in innate host defense. Curr. Pharm. Des..

[7] Strom M.B., Haug B.E., Skar M.L., Stensen W., Stiberg T., Svendsen J.S. (2003). The pharmacophore of short cationic antibacterial peptides. J. Med. Chem..

[8] Mensa B., Kim Y.H., Choi S., Scott R., Caputo G.A., DeGrado W.F. (2011). Antibacterial mechanism of action of arylamide foldamers. Antimicrob. Agents Chemother..

[9] Tew G.N., Scott R.W., Klein M.L., Degrado W.F. (2010). De novo design of antimicrobial polymers, foldamers, and small molecules: From discovery to practical applications. Acc. Chem. Res..

[10] Beckloff N., Laube D., Castro T., Furgang D., Park S., Perlin D., Clements D., Tang H., Scott R.W., Tew G.N. (2007). Activity of an Antimicrobial Peptide Mimetic against Planktonic and Biofilm Cultures of Oral Pathogens. Antimicrob. Agents Chemother..

[11] Hua J., Yamarthy R., Felsenstein S., Scott R.W., Markowitz K., Diamond G. (2010). Activity of antimicrobial peptide mimetics in the oral cavity: I. Activity against biofilms of *Candida albicans*. Mol. Oral Microbiol..

[12] Ryan L.K., Freeman K.B., Masso-Silva J.A., Falkovsky K., Aloyouny A., Markowitz K., Hise A.G., Fatahzadeh M., Scott R.W., Diamond G. (2014). Activity of potent and selective host defense Peptide mimetics in mouse models of oral candidiasis. Antimicrob. Agents Chemother..

[13] Menzel L.P., Chowdhury H.M., Masso-Silva J.A., Ruddick W., Falkovsky K., Vorona R., Malsbary A., Cherabuddi K., Ryan L.K., DiFranco K.M. (2017). Potent in vitro and in vivo antifungal activity of a small molecule host defense peptide mimic through a membrane-active mechanism. Sci. Rep..

[14] Scott R.W., Tew G.N. (2017). Mimics of Host Defense Proteins; Strategies for Translation to Therapeutic Applications. Curr. Top. Med. Chem..

[15] Liu D., Choi S., Chen B., Doerksen R.J., Clements D.J., Winkler J.D., Klein M.L., DeGrado W.F. (2004). Nontoxic membrane-active antimicrobial arylamide oligomers. Angew. Chem. Int. Ed. Engl..

[16] Tew G.N., Liu D., Chen B., Doerksen R.J., Kaplan J., Carroll P.J., Klein M.L., DeGrado W.F. (2002). De novo design of biomimetic antimicrobial polymers. Proc. Natl. Acad. Sci. USA.

[17] Sanati H., Ramos C.F., Bayer A.S., Ghannoum M.A. (1997). Combination therapy with amphotericin B and fluconazole against invasive candidiasis in neutropenic-mouse and infective-endocarditis rabbit models. Antimicrob. Agents Chemother..

[18] Pappas P.G., Kauffman C.A., Andes D.R., Clancy C.J., Marr K.A., Ostrosky-Zeichner L., Reboli A.C., Schuster M.G., Vazquez J.A., Walsh T.J. (2016). Clinical Practice Guideline for the Management of Candidiasis: 2016 Update by the Infectious Diseases Society of America. Clin. Infect. Dis..

[19] Vande Velde G., Kucharikova S., Schrevens S., Himmelreich U., Van Dijck P. (2014). Towards non-invasive monitoring of pathogen-host interactions during *Candida albicans* biofilm formation using in vivo bioluminescence. Cell. Microbiol..

